# Topical and Intranasal Analgesic Therapy in a Woman with Refractory Postherpetic Neuralgia

**DOI:** 10.1155/2015/392874

**Published:** 2015-04-09

**Authors:** Kenneth C. Hohmeier, Lyndsey M. Almon

**Affiliations:** ^1^Department of Clinical Pharmacy, University of Tennessee Health Science Campus, 193 Polk Avenue, Suite 2D, Nashville, TN 37210, USA; ^2^University of Tennessee Health Science Campus, 193 Polk Avenue, Suite 2D, Nashville, TN 37210, USA

## Abstract

A patient-specific, stepped approach to topical and intranasal analgesic pharmacotherapy was effective in reducing refractory postherpetic neuralgia (PHN) not responding to the current standard of care for PHN. The use of topical analgesic therapy allowed for higher concentrations of medication locally while reducing the likelihood of systemic side effects common to the drugs used. No adverse effects were noted for either topical or intranasal drug therapy. The patient-specific, stepped approach resulted in clinically significant decreases in pain on visual analog scale (VAS), with the use of intranasal ketamine 10% solution and topical gabapentin 6%, ketoprofen 10%, lidocaine 5%, and ketamine 10% cream.

## 1. Introduction

New estimates report nearly 16 million people in the United States suffer from neuropathic pain [1, 2], defined as pain due to a damaged peripheral or central nerve [3] caused by a primary lesion or dysfunction in the nervous system [4]. Of these 3.8 million individuals, as many as 1 million suffer from postherpetic neuralgia (PHN) [5], a chronic complication of herpes zoster infection. Commonly referred to as shingles, herpes zoster results from the reactivation of the varicella-zoster virus usually contracted during childhood in the form of the chickenpox [6, 7]. The virus that had remained dormant in a sensory neuron remanifests as a painful, blistering, and vesicular rash along a dermatome [8] or an area of skin innervated by a single spinal nerve [6].

The reported prevalence of herpes zoster ranges between 1.5 and 4 cases per 1000 persons annually [9]. However, this frequency increases at the age of 50 years and older [8, 10], and over 50% of herpes zoster cases occur in persons ≥60 years [9]. By age 90, the incidence rate is as high as 11 cases per 1000 people [7, 10], with a lifetime risk of developing the disease approximated at 30% [6]. The recurrence rate of an additional herpes zoster infection has not been clearly established, yet it is generally perceived to be similar to the rates of a first herpes zoster occurrence. Tseng et al. estimated the incidence rate to be significantly lower than first-occurrence rates at 7.48 cases per 1000 person-years in immunocompetent patients [11], while Yawn et al. report an estimated overall 8-year recurrence rate as high as 6.2% in a mixed immunocompetent and immunocompromised study group [12].

While the hallmark lesioned rash of herpes zoster usually clears within 2 to 4 weeks, 20% of people may experience pain in the form of PHN that persists after the rash has healed [6, 7]. The pain is a consequence of peripheral nerve damage caused by the herpes zoster attack [13]. The duration of pain required for a diagnosis of PHN varies greatly from 1 to 9 months after the onset of the rash [6, 14] and can persist for years. In fact, 15% of patient report pain at 2 years [7]. Reported risk factors for PHN include advanced age, female gender, chronic disease, immunocompromised condition, and a greater severity of outbreak and pain during the acute phase [6, 7].

The pain experienced in PHN is often refractory to therapy, with as many as half of patients failing to respond to any treatment; other patients may experience limited efficacy despite being on multiple agents [6]. Treatment options to date have mainly been centered on oral therapies, including tricyclic antidepressants (TCAs), opioid analgesics, corticosteroids, and anticonvulsants (gabapentin, pregabalin). Therapeutic doses of these oral medications often carry with them a high risk of adverse effects and even addiction. Other current treatment options for PHN already include topical regimens, like lidocaine and capsaicin [7].

According to White et al., herpes zoster patients with PHN have a mean of 17.1 prescriptions filled compared to 5.5 prescriptions for patients without PHN [15]. With limited effective treatments, complicated dosing titration schedules, and overall low patient satisfaction, a gap in care is evident for this patient population [16]. It has been suggested in the literature that a topical, multimodal stepped-care approach to treating refractory pain should be considered [17]. This paper is the first case report discussing the use of such a stepped, patient-specific approach to treatment using a multimodal topical compounded analgesic cream in combination with intranasal ketamine to successfully reduce pain associated with refractory PHN.

## 2. Case Report

On July 26, 2012, a 78-year-old African American woman was referred to our pharmacotherapy consult service with a diagnosis of refractory postherpeticneuralgia (PHN) of the head and neck. This was a recurrent episode of PHN following an original diagnosis in 2010. Pain was noted along the right sternocleidomastoid and masseter muscles per the referring physician. Insomnia and difficulty in speech secondary to pain were also noted. Other relevant medical conditions were rheumatoid arthritis and bilateral osteoarthritis of the knees. Past surgical history included bilateral knee replacements.

Medication history included previous treatment with oral amitriptyline and topical capsaicin 0.025%, neither of which was tolerated by the patient and had been consequently discontinued. Oral amitriptyline was discontinued due to cognitive dysfunction and fatigue. Topical capsaicin was not tolerated due to local pain and discomfort on application of the cream. Her current medication regimen consisted of oxycodone, 20 mg by mouth twice daily, gabapentin, 300 mg by mouth three times daily, oxcarbazepine, 150 mg by mouth three times daily, and tocilizumab administered intravenously using weight-based dosing once monthly. Further increases in titration to the oral gabapentin and oxcarbazepine were not possible due to patient-reported adverse effects.

When the patient presented to our pharmacotherapy consult service, she reported intermittent neuropathic pain that “comes and goes” over the past 2-3 years. According to the patient, this most recent exacerbation had been the worst in her memory. The patient described the pain as “electric,” “tingling,” and occurring with “bursts of lightning,” which originated and were concentrated at the base of the jaw and which subsequently radiated up the lateral and posterior right sides of the cranium. She complained of skin and oral mucosal sensitivity, which was painful to the touch, on the inside and outside of her right cheek. The patient further complained of pain on swallowing, which had lead to the inability to eat full meals. She also complained of insomnia due to persistent baseline pain throughout the night. Severe pain symptoms were described as intermittent, lasting from minutes to hours, with several reported episodes of intense pain surrounding baseline pain. Despite the patient's own description of two separate types of pain—a baseline pain and an intermittent severe pain—on a VAS, she reported both types of pain as “10” on a 10-point visual analog scale (VAS). Due to the acutely severe and intense pain and the patient's severe decline in quality of life, it was decided that the addition of a lidocaine 5% patch alone would likely be insufficient for pain relief, although in most situations a trial of topical lidocaine 5% would have been the recommended next step in treatment. The treating team decided to continue her baseline oral medications and also to initiate immediate treatment with a compounded combination topical analgesic cream composed of gabapentin 6%, ketoprofen 10%, amitriptyline 2%, and lidocaine 5%. The team's anecdotal successful experiences with this combination on other neuropathic pain syndromes led to this initial step. Patient directions were to apply sparingly to the face and neck at the site of pain no more than four times daily, avoiding the area around the eyes. Follow-up was to be conducted by the pharmacy team three days following the initiation of treatment and conveyed to the prescribing physician. Due to the time needed to compound the topical cream, the patient did not begin treatment until the 27th of July.

At the first patient follow-up on the 30th of July, the patient reported a decrease in baseline pain to 7/10 on a VAS; however, peak pain remained constant at 10/10. The patient described her baseline pain as “quieted down” on cream but complained that pain “flare-ups” still remained a problem. At this point, the referring physician and consulting pharmacist made a change to a new topical analgesic cream formulation of gabapentin 6%, ketoprofen 10%, lidocaine 5%, and ketamine 10%. Topical ketamine replaced the amitriptyline due to the patient's concern of side effects based on her previous experience with the amitriptyline oral preparation. An oral mucosal topical analgesic gel was added as well. The antineuropathic agent used was again gabapentin in 10% concentration compounded with commercially available Orabase (a gel with benzocaine 20%). Follow-up with the consulting pharmacist was scheduled for one week after the initiation of the new treatment.

At the next follow-up, the patient reported further decreases to the overall pain. The patient described pain as being at a tolerable level, which allowed her to sleep through the night and 5/10 on a VAS. However, breakthrough pain was still reported to be 8/10. The previous topical regimens were continued, and the addition of a ketamine 100 mg/mL (10%) metered-dose intranasal spray delivering 0.1 mL/spray was initiated. Patient directions were to inhale 1 spray (0.1 mL), alternating nostrils 90 seconds apart, up to three times daily (with a maximum of 5 sprays per dose) for breakthrough pain. The patient was asked to lie in a supine position with her neck extended at a 45-degree angle and to maintain this position for 30 seconds after administration. This dosing recommendation was based on previously successful intranasal ketamine therapy already reported in the literature [18].

Two weeks later, the patient reported further reduction in pain both at rest and when speaking, eating, and drinking. Breakthrough pain was managed on the intranasal ketamine regimen, and an overall reduction in baseline pain to 4/10 was experienced with combination, multimodal pain treatment. The patient reported breakthrough pain relief on intranasal ketamine within 2–5 minutes after administration, on average 2–4 sprays (0.2–0.4 mLs) of the solution. Throughout the duration of therapy, no adverse effects were reported for either the nasal or topical therapies. Therapy was continued for the next several months until full, spontaneous remission of pain.

On November 7, 2013, the patient requested a refill of all medications secondary to a “flare-up.” Due to the patient's age and concomitant immunosuppressive therapy with tocilizumab, recurrent PHN was confirmed on examination. This was the patient's third reported recurrence of PHN. The patient's previous three-prescription regimen was ordered. Again the patient reported a response to treatment, reporting pain score reduction on the VAS from 10/10 to 5/10.

## 3. Discussion

Treatment for postherpetic neuralgia (PHN) is based exclusively on symptom control and targeting the mechanisms causing pain. Oral treatment for PHN can typically include tricyclic antidepressants (TCAs), opioids analgesics, and anticonvulsants. TCAs block sodium, calcium, and NMDA receptors and inhibit serotonin and norepinephrine reuptake [19]. Opioids are mu-receptor agonists and have been shown to alleviate certain neuropathies [13, 20]. Raja et al. investigated these two oral agents by comparing TCAs (nortriptyline 89 mg or desipramine 63 mg) and opioids (morphine 91 mg or methadone 15 mg) and placebo. In his screening of 103 patients, he found that TCAs decreased pain by 32%, while opioids faired mildly better with a 38% improvement. Side effects of both TCAs and opioids are most often cited as reasons for cessation of treatment [21]. Gabapentin and pregabalin treat neuropathies by binding and blocking subunits of calcium channels in neurons, although systemic side effects often limit their use [22]. A recent systematic review of oral gabapentin showed that doses between 1800 and 3600 mg daily provided significant improvement of PHN versus placebo [23]. However, Johnson et al. [24] conducted a retrospective claims database study of 1645 patients to examine real-world treatment with gabapentin (*N* = 939) and pregabalin (*N* = 706) for PHN. He found that over 50% of the patients switched from their original therapies in the treatment observation period of 12 months. Over one-third of those 50% of patients (35% gabapentin, 31% pregabalin) switched to an opioid-based regimen. Also, 37% of the gabapentin index group and 31% of the pregabalin index group added medications to their regimens [24]. Oxcarbazepine monotherapy has also been investigated via an 8-week trial in 24 patients at a maintenance dose of 900 mg orally per day and was shown to significantly reduce the mean VAS score, although mild to moderate side effects were noted [25].

Two commercially available topical agents have been investigated for the treatment of PHN. Both lidocaine and capsaicin are attractive options because their topical route of administration produces relatively low side-effect profiles and low systemic absorption [7]. Topical capsaicin affects the TRPV1 receptor, eventually leading to depletion of substance P, a neurotransmitter known to cause pain and inflammation, from sensory neurons [14, 26]. Topical lidocaine blocks voltage-gated sodium channels [14]. However, like the oral agents, efficacy of topical agents has also been unimpressive. Meier et al. did find a statistically significant decrease in pain with a 5% lidocaine patch, but any conclusions may be questionable as the placebo group reached a significant decrease in pain as well [27]. Capsaicin 0.075% decreased pain marginally by a maximum of 23–30% [28, 29]. The burning adverse reaction on application may limit this treatment, although pretreatment application of lidocaine cream may diminish this side effect.

Our hypothesis was that treatment with a stepped approach using patient-specific, multimodal topical therapy aimed at these pharmacological targets would achieve a reduction in reported pain on a VAS without the adverse effects common in oral therapies. This case report illustrates the use of a stepped approach to refractory PHN using a multimodal topical cream and single-ingredient ketamine intranasal solution. A review of the published evidence for topical therapy in PHN guided our stepped approach. The use of topical NSAIDS and depolarizing anesthetics, such as lidocaine and benzocaine, has been previously investigated in postherpetic nerve pain [30, 31]. Topical gabapentin has been shown to be effective for PHN at concentrations of 6% [32].

Amitriptyline used topically has some limited evidence of effectiveness in the treatment of localized pain [19, 33]. Although we were unable to use amitriptyline and ketamine in combination with this patient, several studies have demonstrated effectiveness with this combination when used topically for a variety of pain conditions [34–36]. Additionally, the pool of evidence for topical ketamine, as both a single agent and in combination with other topical agents, for neuropathic pain continues to grow [19, 36]. It should also be noted that, in this case study, the ketamine used in all formulations was the bulk powder of the racemic form of the active pharmaceutical ingredient (Letco Medical, Decatur, AL).

The decision to add intranasal ketamine was made based on two studies: one study reporting a metered-dose intranasal spray with lidocaine used for trigeminal neuralgia and the other reporting safety and efficacy of intranasal ketamine for the treatment of breakthrough pain [18, 37]. Based on this literature, it was theorized that intranasal delivery of antineuropathic therapy could be successfully delivered to the trigeminal nerve and that ketamine could be used safely and effectively for breakthrough pain; both criteria are necessary for treating our patient effectively. As the patient's symptoms were similar to those seen in patients with trigeminal neuralgia, it seemed a logical target for this patient's refractory breakthrough pain. Anatomically, analgesics administered intranasally should target the trigeminal nerve via the mucosa of the middle turbinate. Therefore, it is likely that the effectiveness seen in our patient may be due to local effects as well as systemic effects.

To date, there have been no reports in the literature regarding the use of intranasal ketamine in patients with PHN. Treatment of PHN using NMDA antagonists has been described previously in the literature via the intravenous route of administration [38]. However, its use in noncancer pain via the rapid-acting intranasal route remains controversial because of its potential risk for addiction [39]. Despite this concern, a double-blind, placebo-controlled crossover trial evaluated the use of intranasal ketamine or placebo in 20 patients with chronic cancer and noncancer pain. Patients in the trial reported significantly lower breakthrough pain (*P* < 0.0001) with intranasal ketamine [18]. Onset of action was 10 minutes on average, with a duration of 60 minutes. Adverse reactions included transient dysgeusia, nasal irritation, rhinorrhea, and increases in blood pressure. No reports of visual or auditory hallucinations were noted on patient questionnaires.

This is the first case report describing the use of a multimodal cream and intranasal ketamine for breakthrough pain in the treatment of PHN. This multimodal cream, intranasal ketamine, or the combination of both should be investigated in larger, randomized, and placebo-controlled trials on the treatment of PHN.
